# Adaptation of food guideline messages for the visually impaired in Lima, Peru: a validation experience

**DOI:** 10.17843/rpmesp.2023.404.12973

**Published:** 2023-12-18

**Authors:** Mirko Lázaro-Serrano, César Domínguez-Curi

**Affiliations:** 1 Instituto Nacional de Salud. Lima, Peru. Instituto Nacional de Salud Lima Peru

**Keywords:** Dietary Guidelines, Visual Impairment Persons, Healthy Lifestyle, Focus Groups

## Abstract

This article aims to describe the design and validation process of food guideline messages written in the braille reading and writing system for people with visual impairment. The process involved the following stages: i) design and elaboration of the material containing the food guideline messages, as well as its adaptation to the format; ii) technical validation with representatives of the National Council for the Integration of Persons with Disabilities (CONADIS); and iii) operational validation through focus groups with children, adolescents and adults with visual disabilities who can read braille. The participants agreed on the legibility, clarity and comprehension of the messages and approved the format of the material. Finally, it should be noted that the participation of key informants and people with visual impairment in all stages was important for the development of an informative material with healthy eating messages in braille.

## INTRODUCTION

Visual impairment, partial or total, is defined as the difficulty that some people have to engage in daily activities due to a specific impairment related to a decrease or loss of visual functions or other barriers in that context. These limitations include the absence of auditory signals to replace visual information and the absence of literature or texts in the Braille reading and writing system, which limits access to information and learning, restricting the right to access information that may allow the growth and integral development of people with this disability ^1^. Worldwide, approximately 2.2 billion people are visually impaired or blind ^2^. The population of Latin America is approximately 630 million people and the average prevalence rate of blindness and moderate to severe visual impairment is 0.37% and 1.98%, respectively, which implies that about 15 million people have some degree of visual impairment in this region ^3^. In Peru, of the total number of people with a disability, around 2 million 487 thousand 690 have only one, with 48.3% (1 million 473 thousand 583) having partial or total visual impairment. This type of disability is the most frequent disability in Peru ^4^.

In Peru, the rights of visually impaired persons are protected by the General Law for Persons with Disabilities (No. 29973) with the aim of enabling them to exercise their rights on equal terms. The law guarantees access to and freedom to choose different formats and means of communication, including Braille, and provides the right to receive quality and inclusive education, as well as including them in different daily activities ^5^. In addition, the right to health of persons with disabilities is a cornerstone for their development ^6^^,^^7^, being the adequate nutritional status an important element that ensures the optimal health status; therefore, food and nutrition education are key factors that contribute to the empowerment of both the individual and the community, encouraging the voluntary adoption of healthy eating habits and lifestyles ^8^.

International evidence on nutritional problems indicates that visual impairment is associated with a high body mass index, especially in boys and school-age children, which is attributed to reduced physical activity or bad eating habits. In addition, malnutrition, characterized by thinning, is frequent in women because they consume less than the recommended daily intake. Interestingly, people with visual impairment do not perceive their disability as an obstacle, they believe that lack of motivation and exercise stops them from taking care of themselves ^9^.

On the other hand, inclusive reading materials on health and nutrition adapted to visually impaired populations are scarce. Some examples of this are the following, an educational toolkit on food and nutrition aimed at teenagers and adults developed in Brazil ^10^, an educational booklet in Braille for the promotion of oral health in children called: “My smile looks happy” in an educational institution in Cuba ^11^ and research initiatives in the state of Hidalgo, Mexico, where food product packaging was labeled in Braille, a survey was applied to participants who were visually impaired in order to measure the impact of its use at the time of purchase and to encourage self-sufficiency in its users ^12^.

In Peru, no studies on the adaptation or validation of educational materials in health or nutrition adapted to Braille were found; however, there are experiences from non-conventional literature related to the development of informational materials aimed at people with visual impairment, including instructions for the safe use of electricity, fuel and consumer rights of these services in Braille ^13^.

Thus, in the framework of promoting healthy eating, the National Center for Food, Nutrition and Healthy Living (CENAN) has been disseminating the Dietary Guidelines for the Peruvian Population since 2019 ^14^. However, since there are no informational and/or educational materials suitable for Braille on this topic, a message booklet of the dietary guidelines has been developed. This tool seeks to promote equal opportunities, access to information, as well as to prevent the problem of malnutrition either by deficit or excess in the visually impaired population. Therefore, this study aimed at describing the process of designing and validating the food guide messages written in Braille for visually impaired people.

## PROCESS FOR OBTAINING THE MATERIAL

**Figure 1:**
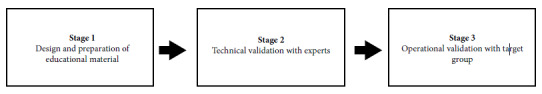
Process for the design-elaboration and validation of educational material for the visually impaired.

This section presents the process, which uses the procedure for the development and validation of technologies in food and nutrition as reference ^15^^,^^16^, and is part of the documentation of the quality management system for educational technologies developed at CENAN. The process consisted of three stages (Figure 1):

### Stage 1: Design and development

During this stage, the technical team conducted a literature review on experiences or interventions for the development of materials suitable for the Braille reading and writing system. Virtual work meetings were held with representatives of the National Council for the Integration of Persons with Disabilities (CONADIS) in order to investigate experiences of previous work in health or nutrition at the national level. Specialists in the design and development of educational materials suitable for Braille were contacted as well.

This staged resulted in a proposed message booklet for the food guides, which considered: i) the contents to be written in Braille, for this, the 12 educational messages of the food guides were used, and a complementary text was added in order to make the messages clearer and more precise for the visually impaired population; and ii) the format of the recommended material to be used for the design and layout was diptych type in A4 size with a minimum font size of 18, considering that there are people with total and partial visual impairment. The quality of the grammage was 180 g in opaline cardboard, as it has the characteristic of greater resistance and durability. The minimum grammage limit is 120 g for Braille printing.

### Stage 2: Technical validation

CONADIS representatives who know and read Braille were included, as well as representatives of the communications area, in order to review and discuss the proposed material, considering criteria such as the typography, the legibility of the text written in Braille and its relevance.

The following procedure was stablished during the work meeting: i) the characteristics of the booklet were presented and explained to the participants, which is a diptych containing 12 educational messages that promote healthy eating, emphasizing that the material was created within the educational contents presented in the food guides for the Peruvian population; ii) the material was handed to the participants giving them a moment to recognize it; and iii) then, each participant was asked to read silently each of the messages, and once finished, CENAN facilitators read aloud, pausing at each of the messages, and then proceeded to ask guiding questions, in order to demonstrate the criteria for understanding the content and relevance of the material.

In this regard, it is important to note the type of typography used during validation, which tries to match the space occupied by the visual text and the Braille text, as well as the size of the font, in macrotype format, since this is an important aspect to promote the inclusion of all types of readers without any extra effort, and enabling the same opportunities to access reading.

The participants considered that the messages were appropriate, they also understood that the content promotes the practice of healthy eating and considered the material to be relevant because materials with information on health or nutrition in Braille are not common. As a result of this stage, a proposal of the booklet to be validated at the operational level was obtained.

### Stage 3: Operational validation

In this stage, prior coordination was made with the authorities of the Luis Braille Alternative Basic Education Center, located in the district of Comas. The objective of the activity was explained, as well as the need to validate the food guide message booklet through focus groups with participants at the primary, secondary and adult levels who are familiar with the Braille reading and writing system. In addition, coordination was made in advance with the authorities of the educational center to inform and request the parents and/or guardians of minors to authorize their participation in the focus groups. The focus groups were conducted by CENAN’s technical team.

The procedure followed during the focus groups had the following sequence: i) the CENAN facilitators introduced themselves and explained the objective of the focus group, which was to review and learn their impressions of the material; ii) the material was handed to each participant, who had a moment to recognize it; iii) participants were asked to read the material in braille; iv) a participant was asked to read aloud the message assigned to him/her in order to corroborate the braille writing with the printed text; and v) the CENAN facilitators read aloud each of the messages again to corroborate with the rest of the participants and, subsequently, the guiding questions were asked, which sought to demonstrate the legibility criteria for braille writing, clarity and comprehension.

Regarding the legibility of the text in Braille, the participants repeatedly emphasized the need to comply with spelling and punctuation rules. Capital letters were adequately used when appropriate and the need to use commas and, especially, the period at the end of each message and its complementary text was emphasized. This stage allowed the visually impaired reader to make a better reading.

Overall, the participants confirmed the clarity and comprehension of the messages; however, it should be noted that in the complementary text of the message referring to ultra-processed foods it was necessary to describe a more precise action (the initial text was modified, which read: “Ask if they have octagons” changing the text to “Ask the seller if they have octagons”) for the identification of products that have the printed front labeling (octagons), therefore, the corresponding modification was made for the final version.

## FINAL VERSION

**Figure 2:**
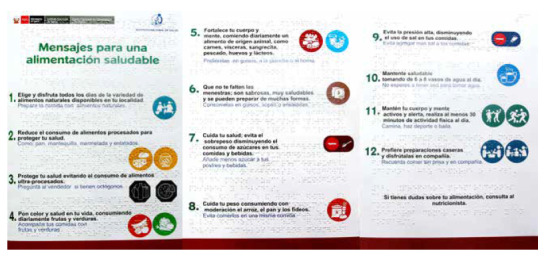
Educational material adapted to Braille system (In its original language, Spanish).

With the information obtained from the technical and operational validation, we proceeded to coordinate with specialists in the design and development of educational and/or informative materials with the Braille reading and writing system to make the corresponding adjustments, resulting in the final version (Figure 2).

## FINAL CONSIDERATIONS

This article describes in detail the steps for the design, development and validation of the booklet titled “Messages for healthy eating”, which is an informative material for tactile exploration of the educational messages of the Peruvian food guides adapted to the Braille system. It should be noted that the resulting material is one of the first in the health sector with inclusive reading adjusted to an alternative format for people with visual impairment, who have limited access to information and health care, which could have adverse consequences for their health and well-being ^17^^,^^18^.

Likewise, the procedure confirms that the inclusion of the target audience was key from the design and elaboration process of the material, especially with the information from CONADIS representatives, in terms of the alternative format, Braille texts and macro types to be used. In addition, during the operational validation process, it was possible to verify the clarity and understanding of the children, adolescents and adults with visual impairment who participated; all this resulted in the final version of the material requiring only minor changes to be resolved when printing the material.

This material is a resource to help in the food and nutrition education that public or private institutions carry out with the visually impaired population in order to ensure the effective exercise of their citizenship ^19^, in addition, it is aligned with the Galway Consensus Statement as it represents a tool that contributes to the promotion of health, which encourages through an innovative material the self-care of visually impaired individuals ^20^.

The process allows establishing a route for the development of other materials with contents of healthy lifestyles suitable for people with visual impairment. In this case, a material printed in the Braille system, but which may also be applied to tools such as: the development of tactile boards with games, voice technologies, audios, among others, in order to establish possible educational interventions in this population with accessible technological resources ^21^^,^^22^.

Finally, the material is a contribution from the National Institute of Health as part of the set of inclusion actions promoted by the Peruvian state such as: the Tiflos service of the National Library of Peru ^23^, which provides free printing of texts at the request of visually impaired people, the Cecograms service, through the Postal Services of Peru - Serpost ^24^, which consists of sending free prints in relief (Braille), sound or numerical records, or special papers for visually impaired people to any destination nationwide.
