# Supplementary material for: Adaptation of food guideline messages for the visually impaired in Lima, Peru: a validation experience
Source: Rev Peru Med Exp Salud Publica. 2023 Dec 18;40(4):485–9. doi: 10.17843/rpmesp.2023.404.12973 (PMC11138831; doi:10.17843/rpmesp.2023.404.12973)
Supplement: Supplementary material. — Available in the electronic version of the RPMESP. [file rpmesp-40-04-12973-s001.pdf]

PERÚ Ministerio de Salud | Instituto Nacional de Salud | Centro Nacional de Alimentación y Nutrición

INSTITUTO NACIONAL DE SALUD

Mensajes para una alimentación saludable

1. Elige y disfruta todos los días de la variedad de alimentos naturales disponibles en tu localidad.
Prepara la comida con alimentos naturales.

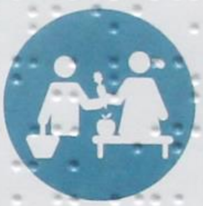

2. Reduce el consumo de alimentos procesados para proteger tu salud.
Como: pan, mantequilla, mermelada y enlatados.

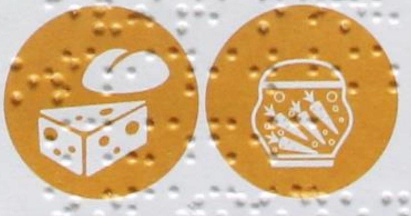

3. Protege tu salud evitando el consumo de alimentos ultra-procesados.
Pregunta al vendedor si tienen octógonos.

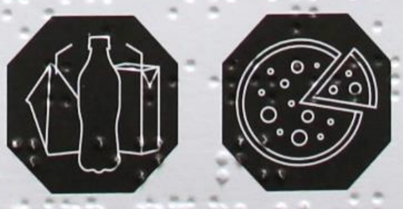

4. Pon color y salud en tu vida, consumiendo diariamente frutas y verduras.
Acompaña tus comidas con frutas y verduras .

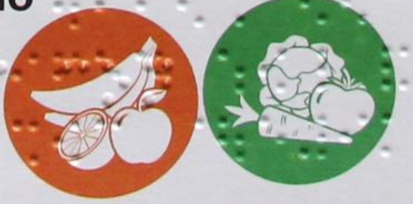

5. **Fortalece tu cuerpo y mente, comiendo diariamente un alimento de origen animal, como carnes, vísceras, sangrecita, pescado, huevos y lácteos.**

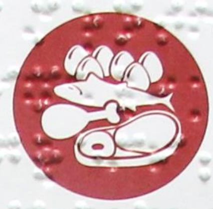
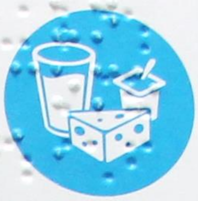

Prefiérelas en guisos, a la plancha o al horno.

6. **Que no te falten las menestras; son sabrosas, muy saludables y se pueden preparar de muchas formas.**

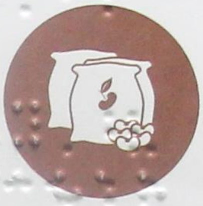

Consúmelas en guisos, sopas o ensaladas.

7. **Cuida tu salud; evita el sobrepeso disminuyendo el consumo de azúcares en tus comidas y bebidas.**

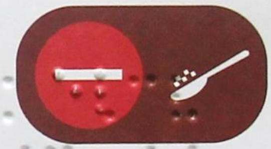

Añade menos azúcar a tus postres y bebidas.

8. **Cuida tu peso consumiendo con moderación el arroz, el pan y los fideos.**

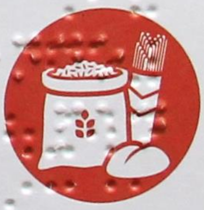

Evita comerlos en una misma comida.

9. **Evita la presión alta, disminuyendo el uso de sal en tus comidas.**
Evita agregar más sal a tus comidas.

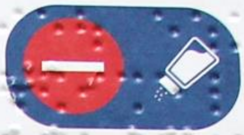

10. **Mantente saludable tomando de 6 a 8 vasos de agua al día.**
No esperes a tener sed para tomar agua.

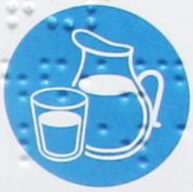

11. **Mantén tu cuerpo y mente activos y alerta, realiza al menos 30 minutos de actividad física al día.**
Camina, haz deporte o baila.

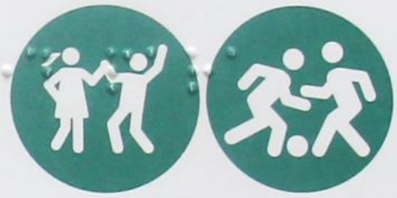

12. **Prefiere preparaciones caseras y disfrútalas en compañía.**
Recuerda comer sin prisa y en compañía.

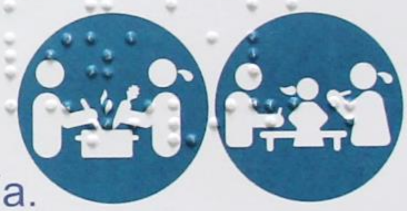

Si tienes dudas sobre tu alimentación, consulta al nutricionista.

INSTITUTO NACIONAL DE SALUD

MINISTERIO DE SALUD

Fuente: Guías alimentarias para la población peruana.
