# Supplementary material for: Adaptation of food guideline messages for the visually impaired in Lima, Peru: a validation experience
Source: Rev Peru Med Exp Salud Publica. 2023 Dec 18;40(4):485–9. doi: 10.17843/rpmesp.2023.404.12973 (PMC11138831; doi:10.17843/rpmesp.2023.404.12973)
Supplement: Translation

# Translation: Adecuación de mensajes de las guías alimentarias para personas con discapacidad visual en Lima, Perú: una experiencia de validación

**Authors:** Mirko Lázaro-Serrano, César Domínguez-Curi

**Affiliations:** 1 Instituto Nacional de Salud. Lima, Perú.

**Keywords:** Guías Alimentarias, Personas con Deficiencia Visual, Estilo de Vida Saludable, Grupos Focales

## Abstract

El objetivo de este artículo es describir el proceso de diseño y validación de los mensajes de las guías alimentarias escritas bajo el sistema de lecto escritura braille dirigido a personas con discapacidad visual. El proceso involucró las siguientes etapas: i) diseño y elaboración del material con el contenido de mensajes de las guías alimentarias, así como, la adecuación del formato; ii) validación técnica con representantes del Consejo Nacional para la Integración de la Persona con Discapacidad (CONADIS); y iii) validación operativa mediante grupos focales con niños, adolescentes y adultos con discapacidad visual que saben leer braille. Los participantes confirmaron la legibilidad, claridad y comprensión de los mensajes y aprobaron el formato del material diseñado. Finalmente, se destaca que la participación de informantes claves y personas con discapacidad visual en todas las etapas resultó relevante para la elaboración de un material informativo con los mensajes de alimentación saludable en braille.

## INTRODUCCIÓN

La discapacidad visual, parcial o total, está definida como la dificultad que presentan algunas personas para participar en actividades de la vida cotidiana y que surge como consecuencia de un impedimento específico relacionado con una disminución o pérdida de las funciones visuales y de las barreras presentes en el contexto. Algunos ejemplos de estas limitaciones son la ausencia de señales auditivas que reemplacen la información visual, ausencia de literatura o textos adecuados al sistema de lecto escritura braille, con lo cual se limita el acceso a la información y aprendizaje tornándose una condición desfavorable para que las personas con esta discapacidad puedan ejercer su derecho pleno de acceder a información que permita un crecimiento y desarrollo integral de la persona ^1^. A nivel mundial, aproximadamente 2200 millones de personas tienen deficiencia visual o ceguera ^2^. En América Latina, con una población aproximadamente de 630 millones de personas, se reportó una prevalencia promedio de ceguera de 0,37% y de discapacidad visual moderada a severa de 1,98%, lo que implica que existen cerca de 15 millones de personas con algún grado de discapacidad visual en esta región ^3^. En Perú, del total de personas con alguna discapacidad, alrededor de 2 millones 487 mil 690 presentan una sola, siendo el 48,3% (1 millón 473 mil 583) aquellas que presenta dificultad parcial o total de la visión. Cabe señalar que, este tipo de discapacidad representa la mayor proporción en el país ^4^. 

En Perú, los derechos de las personas con discapacidad visual están amparados por la Ley General de la Persona con Discapacidad (N° 29973) con el objetivo de permitirles ejercer sus derechos en igualdad de condiciones. La ley garantiza el acceso y libertad de elección de distintos formatos y medios de comunicación, entre ellos, el braille y brinda el derecho de recibir una educación de calidad e inclusiva, además de, incluirlos en diferentes actividades diarias ^5^. Adicionalmente, el derecho a la salud de las personas con discapacidad se constituye en un pilar esencial para su desarrollo ^6^^,^^7^, siendo el adecuado estado nutricional un elemento importante que asegura un óptimo estado de salud de las personas; por ello, la educación alimentaria y nutricional resulta un factor clave que contribuye al empoderamiento tanto del individuo como de la comunidad, fomentando la adopción voluntaria de hábitos alimentarios y estilos de vida saludables ^8^.

Sobre la problemática nutricional, a nivel internacional, la evidencia indica que la discapacidad visual se asocia con un índice de masa corporal elevado, especialmente en varones y niños en edad escolar, lo cual se atribuye a la reducida actividad física o malas elecciones alimentarias. Además, se observa desnutrición, caracterizada por adelgazamiento, en el caso de mujeres debido a que consumen menos de la ingesta diaria recomendada. Resulta interesante destacar que, las personas con discapacidad visual no perciben su discapacidad como un obstáculo, ellos creen que la falta de motivación y ejercicio los inhibieron de tomar el cuidado por ellos mismos ^9^.

Por otro lado, la adecuación de materiales de lectura inclusiva para poblaciones con discapacidad visual es limitada en el campo de la salud y nutrición, siendo destacados un kit de herramientas educativas sobre la temática de alimentación y nutrición dirigido a adolescentes y adultos elaborados en Brasil ^10^, una cartilla educativa en braille para la promoción de salud bucal en niños «*Mi sonrisa se ve feliz*» en una institución educativa en Cuba ^11^ o iniciativas de investigación en el estado de Hidalgo, México, donde se rotularon envases de productos alimentarios en braille, con la finalidad de medir el impacto de su uso al momento de adquirirlos y fomentar la autosuficiencia en sus usuarios, corroborándose a través de la aplicación de una encuesta a los participantes que presentaban discapacidad visual ^12^.

En Perú, no se encontraron publicaciones relacionadas a la adaptación o validación de materiales educativos en salud o nutrición adecuados al braille; no obstante, existen experiencias desde la literatura no convencional relacionadas con la elaboración de materiales informativos dirigidos a personas con discapacidad visual, entre ellos, instructivos para el uso seguro de la electricidad, combustible y derecho de los consumidores de estos servicios en braille ^13^.

Así, en el marco de promover una alimentación saludable, el Centro Nacional de Alimentación, Nutrición y Vida Saludable (CENAN) viene realizando la difusión de las Guías Alimentarias para la Población Peruana desde el año 2019 ^14^. Sin embargo, dado que no existen materiales informativos y/o educativos adecuados al braille sobre esta temática, se ha desarrollado una cartilla de mensajes de las guías alimentarias. Esta herramienta busca promover la igualdad de oportunidades, el acceso a la información, así como, prevenir la problemática de malnutrición sea por déficit o por exceso en la población con discapacidad visual. Por ello, el objetivo de este artículo es describir el proceso desarrollado para el diseño y validación de los mensajes de los guías alimentarios escritos en braille dirigido a personas con discapacidad visual.

## PROCESO PARA LA OBTENCIÓN DEL MATERIAL

En esta sección se presenta el proceso seguido, que toma como referencia el procedimiento de desarrollo y validación de tecnologías en alimentación y nutrición ^15^^,^^16^ que, forma parte de la documentación del sistema de gestión de la calidad para las tecnologías educativas que se desarrollan en el CENAN. El proceso realizado constó de tres etapas (Figura 1):

**Figura 1:** Proceso para el diseño-elaboración y validación de material educativo dirigido a personas con discapacidad visual.

### Etapa 1: Diseño y elaboración

Durante esta etapa, el equipo técnico realizó la revisión bibliográfica sobre experiencias o intervenciones para la elaboración de materiales adecuados al sistema de lecto escritura braille, asimismo, como parte del proceso se establecieron reuniones de trabajo virtuales con representantes del Consejo Nacional para la Integración de la Persona con Discapacidad (CONADIS) con el propósito de indagar experiencias de trabajos previos en salud o nutrición a nivel del país y también se tuvo contacto con especialistas en el diseño y elaboración de materiales educativos adecuados al braille.

El resultado de esta etapa fue la definición de la propuesta de una cartilla de mensajes de las guías alimentarias, la cual consideró: i) los contenidos a ser escritos en braille, para ello, se utilizaron los 12 mensajes educativos de las guías alimentarias, a los cuales se le añadieron un texto complementario a fin de hacer los mensajes más claros y precisos para la población con discapacidad visual; y ii) el formato del material recomendado a utilizar para el diseño y diagramación fue tipo díptico en tamaño A4 con una fuente de letra mínima de 18, considerando que, entre las personas con discapacidad visual total también están las personas con baja visión. La calidad del gramaje utilizado fue de 180 g en cartulina opalina, ya que tiene como característica acabados de mayor resistencia y durabilidad, siendo permitido un gramaje mínimo 120 g para impresión en braille.

### Etapa 2: Validación técnica

Se coordinó con el CONADIS para contar con la participación de representantes que conozcan y lean el braille y representantes del área de comunicaciones, a fin de que revisen y discutan la propuesta del material, considerando criterios como la tipografía utilizada, la legibilidad del texto escrito en braille y la pertinencia del mismo.

El procedimiento seguido durante la reunión de trabajo tuvo la siguiente secuencia: i) se presentaron y explicaron las características de la cartilla a los participantes, que se constituye en un díptico que contiene 12 mensajes educativos que promueven una alimentación saludable, enfatizando que la elaboración del material se enmarca en los contenidos educativos que presentan las guías alimentarias para la población peruana; ii) se entregó el material a los participantes otorgando un momento para su reconocimiento; y iii) luego, se solicitó a cada participante que pueda leer en silencio cada uno de los mensajes, y una vez concluido, los facilitadores de CENAN realizaron la lectura en voz alta, haciendo pausa en cada uno de los mensajes, para luego proceder a realizar las preguntas orientadoras, con la finalidad de evidenciar los criterios de comprensión del contenido y pertinencia del material.

Al respecto, durante la validación se resaltó el uso de la tipografía utilizada, que intenta coincidir entre el espacio ocupado por el texto visual y el texto en braille, así como, el tamaño de la fuente, en formato macrotipo, ya que este es un aspecto a tener en cuenta para promover la inclusión de todo tipo de lectores sin realizar un esfuerzo extra, y posibilitando las mismas oportunidades para acceder a la lectura.

Los participantes consideraron que los mensajes descritos eran adecuados, comprendieron que el contenido promueve la práctica de una alimentación saludable y que el material resultó pertinente debido a que no son comunes los materiales con información en salud o nutrición adecuadas al braille. Como resultado de esta etapa, se obtuvo la propuesta de la cartilla a ser validada a nivel operativo.

### Etapa 3: Validación operativa

En esta etapa se coordinó previamente con las autoridades del Centro de Educación Básica Alternativa Luis Braille, ubicado en el distrito de Comas. Se explicó el objetivo de la actividad e indicó la necesidad de validar la cartilla de mensajes de las guías alimentarias mediante grupos focales con participantes del nivel de primaria, secundaria y adultos que conozcan el sistema de lecto escritura braille. Además, se coordinó anticipadamente con las autoridades del centro educativo a fin de que informen y soliciten a los padres y/o apoderados de menores de edad, la autorización para su participación en los grupos focales. Estos grupos focales fueron conducidos por el equipo técnico del CENAN.

El procedimiento seguido durante los grupos focales tuvo la siguiente secuencia: i) los facilitadores de CENAN se presentaron y explicaron el objetivo del grupo focal, que era revisar y conocer sus impresiones respecto del material; ii) se entregó el material a cada uno de los participantes y se dio un momento para su reconocimiento; iii) se solicitó a los participantes que procedan a leer el material en braille; iv) se solicitó que un participante pueda leer en voz alta el mensaje que se le asignaba a fin de corroborar la escritura en braille con el texto impreso; y v) los facilitadores del CENAN volvieron a leer en voz alta cada uno de los mensajes para corroborarlos con el resto de participantes y, posteriormente, se realizaban las preguntas orientadoras que, buscaban evidenciar los criterios de legibilidad para la escritura en braille, la claridad y la comprensión.

Respecto a la legibilidad del texto descrito en braille, los participantes resaltaron de forma reiterada en la necesidad del cumplimiento de las reglas de ortografía y puntuación. En el caso del primer punto, realizaron un uso adecuado de las mayúsculas donde corresponden, mientras que en el segundo, se remarca la necesidad del uso de las comas y, en especial, el punto aparte y punto final al término de cada mensaje y su texto complementario. Esta etapa permitió al lector con discapacidad visual hacer una mejor lectura.

En general, los participantes confirmaron la claridad y comprensión de los mensajes, sin embargo, es preciso señalar que en el texto complementario del mensaje referido a los alimentos ultra procesados fue necesario describir una acción más precisa (se modificó el texto inicial, el cual decía: «Pregunta si tienen octógonos» cambiándose el texto por «Pregunta al vendedor si tienen octógonos») para la identificación de productos que tienen el etiquetado frontal impreso (octógonos), por ello, se hizo la modificación correspondiente para la versión final.

## VERSIÓN FINAL

Con la información obtenida tanto de la validación técnica y operativa, se procedió a coordinar con los especialistas en el diseño y elaboración de materiales educativos y/o informativos con el sistema de lecto escritura braille para que realicen los ajustes correspondientes, obteniendo la versión final (Figura 2).

**Figura 2:** Material educativo adecuado al sistema braille.

## CONSIDERACIONES FINALES

El presente artículo describe de forma detallada los pasos para el diseño, elaboración y validación de la cartilla denominada «Mensajes para una alimentación saludable» que, es un material informativo de exploración táctil sobre los mensajes educativos de las guías alimentarias peruanas adecuado al sistema braille. Es necesario resaltar que, el material realizado es uno de los primeros en el sector salud con lectura inclusiva ajustada a un formato alternativo para personas con discapacidad visual, que tienen un acceso limitado a información y atención en salud, lo que podría tener consecuencias adversas para su salud y bienestar ^17^^,^^18^.

Asimismo, la actividad conducida confirma que la inclusión del público objetivo resultó clave desde el proceso de diseño y elaboración del material, especialmente con la información obtenida de los representantes de CONADIS, en términos del formato alternativo, textos en braille y macro tipos a usar. Además, durante el proceso de validación operativa, se pudo constatar la claridad y comprensión por parte de los niños, adolescentes y adultos participantes con discapacidad visual; todo ello tuvo como resultado que la versión final del material requiera solo cambios menores a ser resueltos a nivel de la impresión del material.

El material se constituye como un recurso de ayuda en la educación alimentaria y nutricional que las instituciones públicas o privadas realicen con población con discapacidad visual a fin de asegurar el ejercicio efectivo de su ciudadanía ^19^, además, está alineado a la Declaración del Consenso de Galway ya que representa una herramienta que contribuye a la promoción de la salud, que alienta a través de un material innovador el autocuidado de los individuos con discapacidad visual ^20^. 

El proceso seguido permite establecer una ruta para el desarrollo de otros materiales con contenidos de estilos de vida saludables adecuados para las personas con discapacidad visual. En este caso, un material impreso en el sistema braille, pero también, posible de aplicar en herramientas como: el desarrollo de tableros táctiles con juegos, tecnologías de voz, audios, entre otros, a fin de establecer posibles intervenciones educativas en esta población con recursos tecnológicos accesibles ^21^^,^^22^.

Finalmente, el material se constituye en una contribución del Instituto Nacional de Salud como parte del conjunto de acciones de inclusión que promueve el estado peruano tales como: el servicio Tiflos de la Biblioteca Nacional del Perú ^23^ que realiza la impresión gratuita de textos a solicitud de personas con discapacidad visual, el servicio de Cecogramas, a través de los Servicios Postales del Perú - Serpost ^24^, que consiste en el envío gratuito de impresiones en relieve (braille), registros sonoros o numéricos, o papel especial para personas con discapacidad visual a cualquier destino a nivel nacional.
